# Structural and Immunological Insights into the Lipooligosaccharide of the Marine Bacterium *Kangiella japonica* KMM 3897

**DOI:** 10.3390/md23090345

**Published:** 2025-08-28

**Authors:** Alina P. Filshtein, Vlada S. Belova, Alexandra S. Kuzmich, Lyudmila A. Romanenko, Maxim S. Kokoulin

**Affiliations:** 1 G.B. Elyakov Pacific Institute of Bioorganic Chemistry, Far Eastern Branch, Russian Academy of Sciences, 159/2, Prospect 100 let Vladivostoku, Vladivostok 690022, Russia; alishichka@mail.ru (A.P.F.); vladabelova306@gmail.com (V.S.B.); assavina@mail.ru (A.S.K.); lro@piboc.dvo.ru (L.A.R.); 2 Advance Engineering School “Institute of Biotechnology, Bioengineering and Food Systems”, Far Eastern Federal University, 10 Ajax Bay, Russky Island, Vladivostok 690922, Russia

**Keywords:** marine bacteria, *Kangiella japonica*, lipooligosaccharide

## Abstract

The lipooligosaccharide (LOS) of the marine bacterium *Kangiella japonica* KMM 3897 was structurally characterized using chemical analysis, NMR spectroscopy, and MALDI-TOF mass spectrometry. The oligosaccharide core consists of a monophosphorylated trisaccharide containing 2-amino-2-deoxy-D-glucose, D-*glycero*-D-*manno*-heptose, and 3-deoxy-D-*manno*-oct-2-ulosonic acid. The penta-acylated lipid A moiety features a glucosamine disaccharide backbone with phosphate groups and amide- and ester-linked primary fatty acids [i11:0 (3-OH)], along with a secondary acyl chain (i11:0 or 11:0). Immunostimulatory assays revealed that *K. japonica* KMM 3897 LOS induced significantly weaker cytokine production in human peripheral blood mononuclear cells (PBMCs) compared with *E. coli* LPS. Notably, it exhibited potent antagonistic activity against *E. coli* LPS-mediated toxicity and suppressed caspase-4 activation in LPS-treated PBMCs. These findings highlight its anti-inflammatory and protective properties.

## 1. Introduction

Marine environments are a treasure trove of microbial diversity, harboring some of the most ancient and resilient life forms on Earth. Over millions of years, marine microorganisms have evolved sophisticated mechanisms to thrive in dynamic and extreme oceanic conditions, including frigid temperatures, fluctuating salinity, and immense pressure [1,2,3]. These harsh environmental factors have driven the development of unique survival strategies, particularly in the structural components of microbial cells. A prime example is the cell envelope, the critical interface between the microorganism and its surroundings. Constantly exposed to environmental stressors, the cell envelope must maintain precise organization of its key components to ensure physiological stability and functionality under extreme conditions while remaining flexible enough to adapt to the ever-shifting marine environment. This delicate balance between stability and adaptability underscores the complexity of microbial life in the oceans, highlighting the importance of understanding the molecular mechanisms underlying their survival and ecological success [1,2,3].

Among marine microorganisms, Gram-negative bacteria are widespread, thriving in diverse habitats ranging from the deep sea to coastal waters and from free-living forms to symbiotic relationships with other organisms [1]. Their extraordinary adaptability is evident in their extensive phylogenetic diversity, which exceeds that of bacteria in terrestrial environments. Marine Gram-negative bacteria have evolved diverse strategies to thrive in these highly variable and frequently extreme conditions, many of which involve structural adaptations within their cell envelope components. Central to these adaptations are lipopolysaccharide (LPS) molecules, which play a pivotal role in protecting against external threats while maintaining cellular homeostasis [4]. Structurally, LPS consists of three domains: lipid A, the core oligosaccharide, and the O-polysaccharide. When the O-polysaccharide is absent, the LPS is termed rough-type lipopolysaccharide (R-LPS) or lipooligosaccharide (LOS), while its presence designates it as smooth-type LPS (S-LPS) [4,5]. The O-polysaccharide, known for its high variability, is vital for bacterial survival in marine environments. Lipid A and core oligosaccharide, though less variable, still undergo significant structural modifications to enhance membrane stability and resilience. These adaptations not only enable survival in extreme conditions but also provide insights into the evolutionary strategies of marine bacteria [4].

Beyond their role in microbial adaptation, LPS molecules are involved in host–microbe interactions, including colonization, virulence, and symbiosis. In mammals, LPS is recognized by the toll-like receptor 4 (TLR4)/myeloid differentiation protein-2 (MD-2) complex, triggering immune responses [6,7,8,9]. Upon binding to lipid A, this complex activates downstream signaling pathways, including nuclear factor-kB (NF-kB), leading to the release of proinflammatory mediators. While this response is essential for combating infections, its overactivation can result in life-threatening conditions such as sepsis and septic shock.

In addition to its well-characterized interactions with TLR4/MD-2, LPS can form complexes with cytosolic structures to amplify the host immune response. A striking example is its direct binding to inflammatory caspases, which act as intracellular sentinels for LPS detection. Upon cytosolic entry, LPS is specifically recognized by human caspase-4 and caspase-5 (or caspase-11 in mice). These receptors exhibit high affinity and selectivity for LPS, initiating the assembly of the noncanonical inflammasome (NLRP3). Mechanistically, LPS binding induces the oligomerization of caspase-4 monomers, unleashing their proteolytic activity. Activated caspase-4/5/11 triggers NLRP3 inflammasome activation, leading to caspase-1-dependent processing and secretion of IL-1β and IL-18, as well as the induction of pyroptotic cell death, a critical driver of endotoxin-mediated pathology [9,10,11].

However, the immunostimulatory potential of LPS is highly structure-dependent, with some LPS variants acting as potent receptor agonists and others exhibiting weak or antagonistic properties. Indeed, structural variations in lipid A, including differences in acylation patterns, fatty acid length and composition, and phosphate group modifications, are closely linked to its diverse immunological effects [6,7,8,9,10,11,12,13,14,15]. Furthermore, subtle variations in the LPS core structure influence not only TLR4/MD-2 binding but also caspase-4 binding [16,17,18].

This variability has sparked interest in LPS as a source of immunomodulatory compounds, particularly those with unique structural features that could serve as receptor antagonists. Marine bacteria synthesize LPS molecules with distinct chemical properties, some of which exhibit desirable characteristics. These unique LPS structures offer significant potential for modulating immune responses and advancing the development of innovative therapeutics, especially for treating conditions such as endotoxic shock [4,5,6,7,8,9]. Consequently, the structural elucidation of lipid A and other LPS components from marine bacteria is essential for understanding microbial adaptation and inspiring the development of natural-source immunotherapeutics.

This study focused on the LOS derived from *Kangiella japonica* KMM 3897, a marine bacterium isolated from a coastal seawater sample collected in the Sea of Japan [19]. Previous research revealed that bacteria of the genus *Kangiella* produce unique cell-wall-associated polysaccharides [20,21]. These polysaccharides demonstrated a selective antiproliferative effect against human breast T-47D cells, highlighting their potential biomedical relevance [20,21,22]. This paper delves into the structural characterization of the lipid A and core oligosaccharide components of the LOS from *K. japonica* KMM 3897. Additionally, the proinflammatory activity of this LOS in human peripheral blood mononuclear cells (PBMCs) was investigated to elucidate its biological effects and potential implications for modulating the immune response.

## 2. Results

### 2.1. LOS Extraction, Purification, and Characterization

Dried cells of *K. japonica* KMM 3897 were subjected to extraction using the PCP method [23], followed by enzymatic treatment and ultracentrifugation. The obtained preparation, when analyzed by silver-stained SDS-PAGE (Figure S1), exhibited a predominant low-molecular-mass smear at the bottom of the gel, indicative of the R-form LPS (LOS) [20]. Additionally, a minor ladder-like pattern observed in the middle of the gel suggested the co-presence of S-form LPS. Similar to *K. japonica* KMM 3899^T^ [20], the O-polysaccharide portion of the LPS from *K. japonica* KMM 3897 shares an identical structure with the capsular polysaccharide previously characterized [21]. This structural similarity was confirmed through ^1^H NMR analysis (Figure S2), which revealed consistent chemical shifts between the two polysaccharides.

Analysis of the total fatty acid content in the LPS preparation revealed the presence of 3-hydroxyisoundecanoic acid [i11:0 (3-OH)], undecanoic acid (11:0), and isoundecanoic acid (i11:0) in a ratio of 3.8:1.1:1 (detector response). To obtain detailed compositional and structural information on the oligosaccharide components, the LPS underwent complete deacylation, followed by separation using gel-permeation chromatography. Given that the O-polysaccharide of the LPS contains uronic acids, which are subject to decomposition by the β-elimination mechanism, the resulting fragments included the core oligosaccharide and the carbohydrate backbone of lipid A, the latter featuring a glycosyl phosphate group at the reducing end that remains stable under alkaline conditions. Monosaccharide analysis of the oligosaccharide (OS) preparation, conducted by gas chromatography–mass spectrometry (GC-MS) of acetylated methyl- and (*S*)-2-butyl glycosides, indicated the presence of 2-amino-2-deoxy-D-glucose (D-GlcN) and D-*glycero*-D-*manno*-heptose (D,D-Hep). Further sugar analysis, following treatment of the oligosaccharides with hydrofluoric acid, revealed the additional presence of 3-deoxy-D-*manno*-oct-2-ulosonic acid (Kdo), suggesting phosphorylation at this residue. Subsequent analysis of the OS provided a representative and detailed characterization of the core oligosaccharide structure.

### 2.2. Structural Elucidation of the Core Oligosaccharide via NMR Spectroscopy

**Figure 1:**
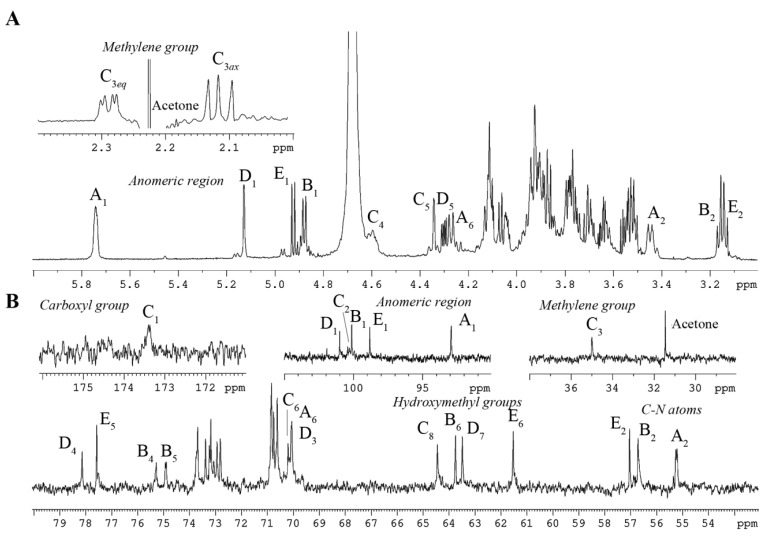
^1^H (**A**) and ^13^C NMR (**B**) spectra of the OS from *K. japonica* KMM 3897. Numerals refer to atoms in sugar residues, which are denoted by capital letters, as outlined in Table 1.

The ^1^H NMR spectrum of the OS (Figure 1A and Table 1) displayed, among other signals, four anomeric proton resonances at δ_H_ 5.74 (^3^J_H1–H2_ 3.3 Hz; H-1 of residue **A**), 5.13 (^3^J_H1–H2_ 1.4 Hz; H-1 of residue **D**), 4.92 (^3^J_H1–H2_ 8.4 Hz; H-1 of residue **E**), and 4.88 (^3^J_H1–H2_ 8.6 Hz; H-1 of residue **B**). Additionally, the signals observed at δ_H_ 2.29 and 2.12 were identified as H-3*eq* and H-3*ax* methylene protons corresponding to the Kdo*p* residue (**C**).

The ^13^C NMR spectrum of the OS (Figure 1B and Table 1) revealed several key carbon signals. Among these, five peaks were observed in the anomeric region at δ_C_ 101.0 (C-1 of residue **D**), δ_C_ 100.3 (C-2 of residue **C**), δ_C_ 100.1 (C-1 of residue **B**), δ_C_ 98.8 (C-1 of residue E), and δ_C_ 92.9 (C-1 of residue **A**). Three signals were detected for carbons bonded to nitrogen atoms at δ_C_ 57.0 (C-2 of residue **E**), δ_C_ 56.7 (C-2 of residue **B**), and δ_C_ 55.2 (C-2 of residue **A**). The spectrum also featured five hydroxymethyl carbon signals at δ_C_ 70.1 (C-6 of residue **A**), δ_C_ 64.4 (C-8 of residue **C**), δ_C_ 63.8 (C-6 of residue **B**), δ_C_ 63.5 (C-7 of residue **D**), and δ_C_ 61.5 (C-6 of residue **E**), and a single methylene group at δ_C_ 35.0 (C-3 of residue **C**) (data from the DEPT-135 experiment; Figure S3). Furthermore, the ^31^P NMR spectrum indicated the presence of three distinct phosphate ester groups, with chemical shifts at δ_P_ −1.69 and 0.25 (double integral intensity).

**Figure 2:**
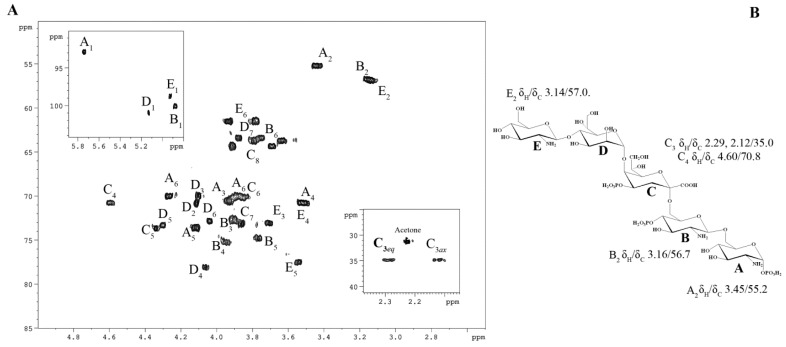
(**A**) ^1^H, ^13^C HSQC spectrum of the OS from *K. japonica* KMM 3897. Numerals refer to atoms in sugar residues, which are denoted by capital letters, as outlined in Table 1. (**B**) OS structure and key cross-peaks.

**Figure 3:**
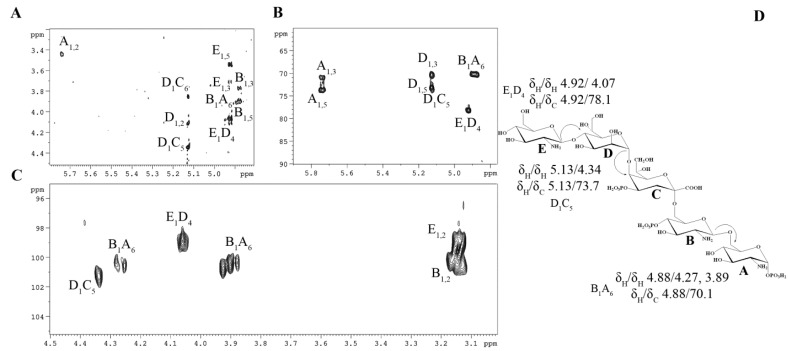
Fragments of ^1^H,^1^H ROESY (**A**) and ^1^H, ^13^C HMBC (**B**,**C**) spectra of the OS from *K. japonica* KMM 3897. Numerals refer to atoms in sugar residues, which are denoted by capital letters, as outlined in Table 1. (**D**) OS structure and key cross-peaks.

**Figure 4:**
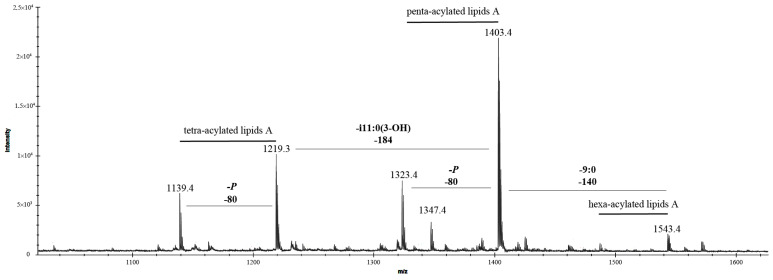
Negative-ion MALDI-TOF mass spectrum of the lipid A from *K. japonica* KMM 3897 recorded in reflector mode. “-i11:0(3-OH)” indicates the lack of a 3-hydroxyisoundecanoic acid, whereas “-P” indicates the lack of a phosphate group.

**Figure 5:**
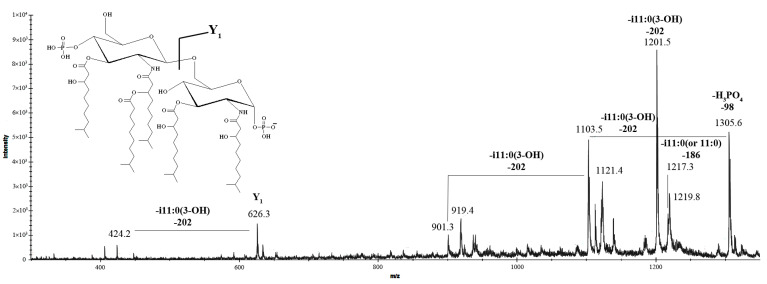
MALDI tandem mass spectrometry (MS/MS) analysis of the penta-acylated lipid A species at *m*/*z* 1403.4 from *K. japonica* KMM 3897. Fragment assignments are indicated. “-i11:0(3-OH)” indicates the loss of a 3-hydroxyisoundecanoic acid, “-i11:0 (or 11:0)” indicates the loss of an isoundecanoic or undecanoic acid, and “-H_3_PO_4_” indicates the loss of a phosphate group. The i11:0 acyl chain is shown only for illustrative purposes; the actual composition may include a linear form as well.

To achieve comprehensive structural elucidation (Table 1), a suite of 2D NMR experiments was employed. Each spin system was assigned through analysis of the ^1^H,^1^H COSY (Figure S4) and ^1^H,^1^H TOCSY (Figure S5) spectra, complemented by the ^1^H,^13^C HSQC (Figure 2) and ^1^H,^13^C HMBC spectra (Figure 3B,C). These experiments collectively identified each proton and carbon atom within the molecule. The anomeric configuration of each hexose and heptose residue was determined by analyzing the ^3^J_H1-H2_ coupling constants. These findings were further corroborated by intra-residual nuclear Overhauser effect (NOE) interactions identified in the ^1^H,^1^H ROESY spectrum (Figure 3A) and C-5 chemical shifts. The precise positioning of the phosphate groups was determined using the ^1^H,^31^P HMBC experiment.

In detail, sugar residues **A** and **B** were identified as the D-Glc*p*N units of the lipid A disaccharide backbone, supported by their H-2/C-2 correlations at δ_H_/δ_C_ 3.45/55.2 and 3.16/56.7 in the ^1^H,^13^C HSQC spectrum (Figure 2), respectively. Both D-Glc*p*N units were phosphorylated, which was supported by the detection of correlations between H-1 of residue **A**, H-4 of residue **B**, and the corresponding phosphate groups at δ_H_/δ_P_ 5.74/−1.69 and 3.95/0.25, respectively (data of the ^1^H, ^31^P HMBC experiment). The assignment of residues **A** and **B** to the lipid A moiety was further validated by the observation of the inter-residue NOE cross-peaks **B** H-1/**A** H-6 at δ_H_/δ_H_ 4.88/4.27, 3.89 (data of the ^1^H,^1^H ROESY experiment) and the long-range scalar correlation **B** H-1/**A** C-6 at δ_H_/δ_C_ 4.88/70.1 (data of the ^1^H,^13^C HMBC experiment; Figure 3B,C). Residue **E** was identified as the Kdo*p* unit, with the assignment beginning with the two methylene protons at δ_H_ 2.29 and 2.12. In the ^1^H,^1^H TOCSY spectrum, these protons were correlated with signals at δ_H_ 4.60 and 4.34, assigned to H-4 and H-5, respectively. The downfield shift of the C-5 signal at δ_C_ 73.7, in contrast with the value observed for the unsubstituted monosaccharide [24], indicated that glycosylation of the Kdo*p* residue occurred at the *O*-5 position. Additionally, the downfield-shifted H-4 and C-4 signals at δ_H_ 4.60 and δ_C_ 70.8, respectively, suggested the phosphorylation of the Kdo*p* residue at this position. This finding was corroborated by the ^1^H,^31^P HMBC spectrum, which displayed a correlation between H-4 and the phosphate group at δ_H_/δ_P_ 4.60/0.25. The α-configuration of the Kdo*p* residue was inferred from the difference (0.17 ppm) between the H-3*eq* and H-3*ax* chemical shifts, and this was further confirmed by the chemical shift of C-6 at δ_C_ 70.2, consistent with the published data [24]. Residue **D** was identified as the α-D,D-Hep*p* unit, which was suggested by the H1/H2 correlation observed in the ^1^H,^1^H TOCSY spectrum, indicating a *manno*-configuration. It was determined that the α-D, D-Hep*p* residue was substituted at the *O*-4 position, as evidenced by the downfield chemical shift of its C-4 compared with the reference value for an unsubstituted residue [25]. Finally, residue **E** was recognized as a *gluco*-configured aminosugar. The ^1^H,^1^H TOCSY experiment showed correlations from the H-1 to H-6 signals, and H-2 was correlated with a carbon linked to a nitrogen atom at δ_H_/δ_C_ 3.14/57.0. This residue was identified as a terminal β-D-Glc*p*N, as no glycosylation shift of its carbons was observed [26].

The sequence of sugar residues was elucidated through a detailed analysis of the ^1^H,^1^H ROESY (Figure 3A) and ^1^H,^13^C HMBC spectra (Figure 3B,C). The lipid A disaccharide backbone, comprising residues **A** and **B**, was identified as the starting point for analysis. Residue **B** was found to be substituted at the *O*-6 position by the Kdo*p* residue (**C**). This substitution was supported by a weak downfield shift of the C-6 signal of residue **B**, characteristic of ketose glycosylation. The Kdo*p* residue (**C**) was further substituted at *O*-5 by the α-D,D-Hep*p* residue (**D**), as confirmed by the correlations H-1 **D**/H-5 **C** at δ_H_/δ_H_ 5.13/4.34 in the ^1^H,^1^H ROESY spectrum, and H-1 **D**/C-5 **C** at δ_H_/δ_C_ 5.13/73.7 in the ^1^H,^13^C HMBC spectrum. Additionally, the α-D,D-Hep*p* residue (**D**) was glycosylated at its *O*-4 position by the β-D-Glc*p*N unit (**E**). This linkage was evidenced by the scalar correlation H-1 E/C-4 **B** at δ_H_/δ_C_ 4.92/78.1 in the ^1^H,^13^C HMBC spectrum and by the NOE contact between H-1 of residue **E** and H-4 of residue **D** at δ_H_/δ_H_ 4.92/4.07 in the ^1^H,^1^H ROESY spectrum.

Based on the obtained data, the structure of the OS is illustrated as follows:

β-D-Glc*p*N-(1→4)-α-D,D-Hep*p*-(1→5)-α-Kdo*p*4P-(2→6)-β-D-Glc*p*N4P-(1→6)-β-D-Glc*p*N1P

### 2.3. MALDI-TOF Mass Spectrometry Analysis of Lipid A

To elucidate the full structure of the glycolipid component and identify the distribution of acyl chains across the glucosamine disaccharide backbone, mild acid hydrolysis of the LPS was carried out. The resulting lipid A fraction was subsequently analyzed using matrix-assisted laser desorption ionization time-of-flight mass spectrometry (MALDI-TOF MS) and MS/MS.

The negative-ion MALDI-TOF mass spectrum of the lipid A revealed three distinct clusters of peaks, each corresponding to unique glycoforms characterized by variations in fatty acid composition and phosphate content (Figure 4). The ion peaks identified at *m*/*z* 1403.4, 1323.4, 1219.3, and 1139.4 were assigned to bis- and mono-phosphorylated penta- and tetra-acylated glycoforms, respectively. Specifically, the peak at *m*/*z* 1403.5 was attributed to a bis-phosphorylated penta-acylated lipid A species containing four i11:0(3-OH) fatty acid units and one i11:0 (or 11:0) unit. Its mono-phosphorylated counterpart was observed at *m*/*z* 1323.4. Additionally, the ion peak at *m*/*z* 1219.3 was identified as a bis-phosphorylated tetra-acylated lipid A species lacking one i11:0(3-OH) residue, while its mono-phosphorylated form was detected at *m*/*z* 1139.4. A minor peak at *m*/*z* 1347.4 was assigned to another bis-phosphorylated penta-acylated lipid A species, which also contained four i11:0(3-OH) fatty acid units and one 7:0 unit (Table 2). Furthermore, a minor peak at *m*/*z* 1543.3 was designated as a bis-phosphorylated hexa-acylated species, distinguished by an additional 9:0 fatty acid unit.

Negative-ion MS/MS analysis was employed to precisely determine the positioning of acyl chains relative to the glucosamine disaccharide backbone of lipid A. To characterize both *inter*- and *intra*-ring fragmentation patterns, the nomenclature proposed by Costello and Vath [27] was applied. Specifically, the MS/MS spectrum of the precursor ion at *m*/*z* 1403.4 (Figure 5) displayed a prominent fragment peak at *m*/*z* 1305.6, corresponding to the loss of the phosphate moiety.

The ion peaks observed at *m*/*z* 1217.3 and 1201.5 were identified as fragments resulting from the elimination of the i11:0 (or 11:0) and i11:0(3-OH) fatty acid chains, respectively. The peak at *m*/*z* 1103.5 was assigned to a fragment lacking the phosphate group and the i11:0(3-OH) acyl unit. Notably, the absence of peaks corresponding to the loss of an *O*-acyloxyacyl moiety in the spectrum suggested that the secondary i11:0 (or 11:0) acyl chain was directly linked to the amide-bound fatty acid. The Y_1_ ion at *m*/*z* 626.3, arising from the cleavage of the glycosidic bond within the glucosamine disaccharide backbone, provided evidence that the proximal α-D-Glc*p*N unit was di-acylated [2 × i11:0(3-OH)], while the distal β-D-Glc*p*N unit was tri-acylated [2 × i11:0(3-OH) and i11:0 (or 11:0)]. Further supporting this structural assignment, the peak at *m*/*z* 424.2 was attributed to the Y_1_ ion after the additional loss of an i11:0(3-OH) acyl chain, confirming the acylation pattern of the glucosamine disaccharide.

**Figure 6:**
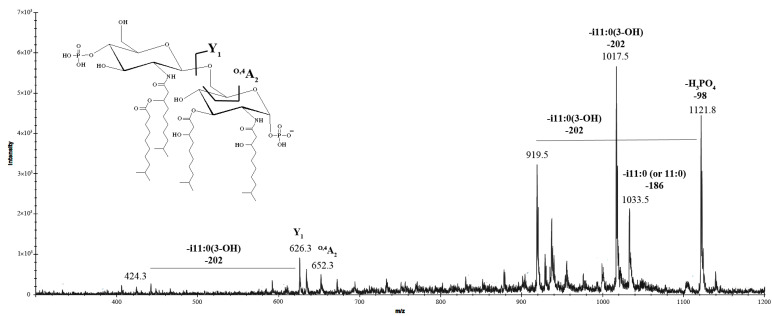
MALDI tandem mass spectrometry (MS/MS) analysis of the tetra-acylated lipid A species at *m*/*z* 1219.3 from *K. japonica* KMM 3897. Fragment assignments are indicated. “-i11:0(3-OH)” indicates the loss of a 3-hydroxyisoundecanoic acid, “-i11:0 (or 11:0)” indicates the loss of an isoundecanoic or undecanoic acid, and “-H_3_PO_4_” indicates the loss of a phosphate group. The i11:0 acyl chain is shown only for illustrative purposes; the actual composition may include a linear form as well.

The negative-ion MS/MS spectrum of the precursor ion at *m*/*z* 1219.3 (Figure 6), corresponding to a bis-phosphorylated lipid A species featuring three i11:0(3-OH) acyl chains and one i11:0 (or 11:0) unit, revealed key fragment ions that provided insights into its structural composition. Prominent peaks at *m*/*z* 1121.8, 1033.5, and 1017.5 were identified as fragments resulting from the loss of the phosphate group, i11:0 (or 11:0), and i11:0(3-OH) acyl chains, respectively. The peak at *m*/*z* 919.5 was assigned to a fragment lacking the phosphate group and i11:0(3-OH) unit. Further structural elucidation was achieved by detecting Y_1_ ions at *m*/*z* 626.3 and 442.1 [corresponding to a loss of 184 amu, i11:0(3-OH)]. These ions confirmed that the proximal α-D-Glc*p*N unit was di-acylated with two i11:0(3-OH) chains, while the distal β-D-Glc*p*N unit, in the tetra-acylated form of lipid A, was di-acylated with one i11:0(3-OH) and one i11:0 (or 11:0) chain. The presence of the ^O,4^A_2_ ion at *m*/*z* 652.3 validated the acylation pattern, conclusively confirming the arrangement of acyl chains on the disaccharide backbone.

The negative-ion MS/MS spectra of the precursor ions at *m*/*z* 1323.4 and 1139.4 revealed a series of successive losses corresponding to acyl chains. The absence of Y_1_ ions indicated that the phosphate group on the proximal α-D-Glc*p*N residue was missing, most likely removed during the hydrolysis process. The presence of the ^O,4^A_2_ ion at *m*/*z* 652.3 in the MS/MS spectra of the precursor ion at *m*/*z* 1139.4 confirmed the acylation pattern of the tetra-acylated form.

Unfortunately, the precise locations of the acyl chains on the minor penta- and hexa-acylated species could not be determined due to their low abundance in the sample and the inability to obtain high-quality MS/MS spectra. Their exact positions remain to be elucidated and confirmed.

**Figure 7:**
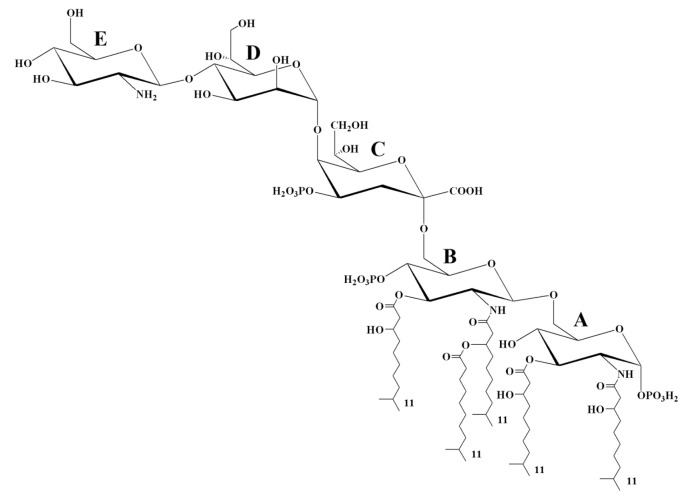
Proposed structure of the LOS from *K. japonica* KMM 3897. The i11:0 acyl chain is shown only for illustrative purposes; the actual composition may include a linear form as well.

Thus, by integrating the results from compositional analysis, NMR spectroscopy, MS, and MS/MS, the structure of the LOS from *K. japonica* KMM 3897 was determined. The proposed structure, with the predominant lipid A species, is illustrated in Figure 7.

### 2.4. Immunological Properties of LOS

The biological activity of LPS molecules, whether agonistic or antagonistic, is determined by the composition and arrangement of their acyl chains on the lipid A moiety. A classic example is the bis-phosphorylated hexa-acylated lipid A from *E. coli*, which demonstrates agonistic activity by engaging the TLR4/MD-2 complex and triggering downstream pro-inflammatory signaling cascades. However, even minor structural alterations within specific domains of lipid A can profoundly modulate its immunostimulatory or immunosuppressive effects. For instance, bis- and mono-phosphorylated tetra- and penta-acylated variants of lipid A are often associated with antagonistic properties, effectively dampening TLR4-mediated immune responses [6,7,8,9]. Notably, structural determinants of LPS dictate its inflammasome-stimulatory capacity. While hexa-acylated LPS robustly promotes caspase-4 oligomerization and activation, hypo-acylated variants—despite binding the caspase activation and recruitment domain (CARD) with comparable affinity—fail to induce oligomerization in vitro [9,10,11].

In this context, given the elucidated structure of the LOS from *K. japonica* KMM 3897, one of the key objectives of this study was to evaluate its capacity to induce an immune response in human blood cells and to assess its ability to modulate the effects of *E. coli* LPS, one of the most extensively studied and potent activators of immune cells.

**Figure 8:**
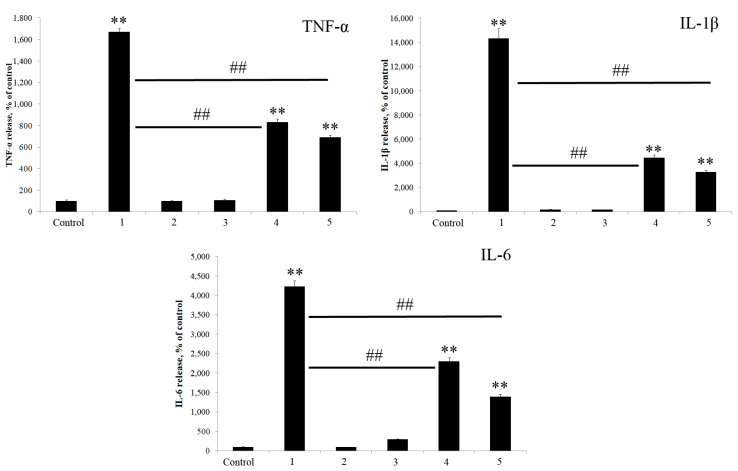
Induction of cytokines in the presence of *E. coli* LPS and *K. japonica* KMM 3897 LOS. PBMCs were stimulated with 10 ng/mL and 100 ng/mL of *K. japonica* KMM 3897 LOS, or *E. coli* LPS at a concentration of 10 ng/mL. In the competition assay, PBMCs were pretreated for 1 h with 10 and 100 ng/mL of *K. japonica* KMM 3897 LOS and then stimulated with *E. coli* LPS at a concentration of 10 ng/mL for 18 h. The results are expressed as the % change in cytokines’ production relative to the negative control (100%). Each bar represents the mean ± SEM of three independent replicates (*n* = 3). Statistical significance was determined by one-way ANOVA followed by Tukey’s Honest Significant Difference test. (**) *p* < 0.01 indicates significant differences from the control group (untreated cells), and (^##^) *p* < 0.01 indicates significant differences from the *E. coli* LPS-treated group. 1—*E. coli* LPS (10 ng/mL), 2—*K. japonica* LOS (10 ng/mL), 3—*K. japonica* LOS (100 ng/mL), 4—*K. japonica* LOS (10 ng/mL) + *E. coli* LPS (10 ng/mL), and 5—*K. japonica* LOS (100 ng/mL) + *E. coli* LPS (10 ng/mL).

We investigated the immunomodulatory effects of *K. japonica* KMM 3897 LOS on human innate immune responses using an in vitro model. To assess its immunopotential, human PBMCs were stimulated for 18 h with *K. japonica* KMM 3897 LOS at concentrations of 10 and 100 ng/mL, alongside *E. coli* LPS at 10 ng/mL as a comparative control. The production of key inflammatory cytokines, TNF-α, IL-1β, and IL-6 was quantified to evaluate immune activation. Notably, stimulation with *K. japonica* KMM 3897 LOS resulted in minimal cytokine release, suggesting a weak immunostimulatory effect. In stark contrast, *E. coli* LPS triggered a robust cytokine response, with significantly higher levels of TNF-α, IL-1β, and IL-6 detected (Figure 8).

Next, we explored the capacity of *K. japonica* KMM 3897 LOS to antagonize the cytokine release induced by *E. coli* LPS, aiming to uncover its potential immunomodulatory properties. To test this, human PBMCs were pre-treated for 1 h with varying concentrations (10 and 100 ng/mL) of *K. japonica* KMM 3897 LOS, followed by stimulation with *E. coli* LPS (10 ng/mL) for 18 h (Figure 8). Strikingly, the results demonstrated that *K. japonica* KMM 3897 LOS effectively suppressed the *E. coli* LPS-driven cytokine response at both concentrations tested (Figure 8). This significant inhibition of pro-inflammatory cytokine release suggests that *K. japonica* KMM 3897 LOS may act as a potent antagonist of TLR4 signaling. All these data indicate that *K. japonica* KMM 3897 LOS acts as a weak immunostimulatory agent on human immune cells and shows good inhibitory activity toward the potent agonist LPS.

**Figure 9:**
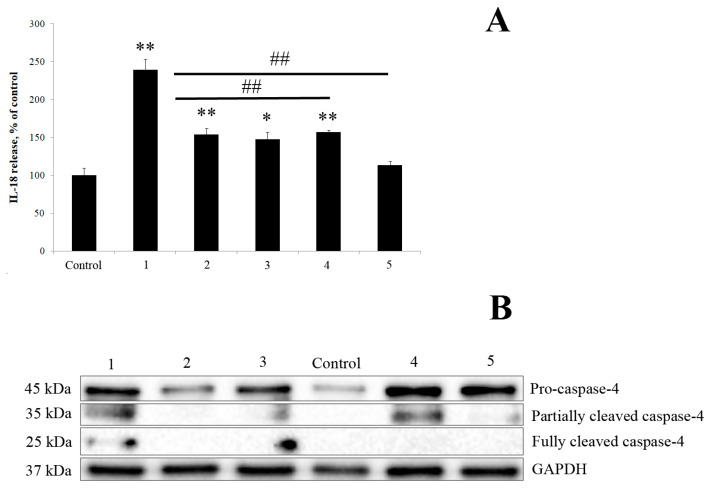
(**A**) Induction of IL-18 in the presence of *E. coli* LPS and *K. japonica* KMM 3897 LOS. PBMCs were stimulated with 1 µg/mL and 10 µg/mL of *K. japonica* KMM 3897 LOS, or *E. coli* LPS at a concentration of 1 µg/mL. In the competition assay, PBMCs were pretreated for 1 h with 1 and 10 µg/mL of *K. japonica* KMM 3897 LOS and then stimulated with *E. coli* LPS at a concentration of 1 µg/mL for 18 h. The results are expressed as the % change in cytokines’ production relative to the negative control (100%). Each bar represents the mean ± SEM of three independent replicates (*n* = 3). Statistical significance was determined by one-way ANOVA followed by Tukey’s Honest Significant Difference test. (*) *p* < 0.05 and (**) *p* < 0.01 indicate significant differences from the control group (untreated cells), whereas (^##^) *p* < 0.01 indicates significant differences from the *E. coli* LPS-treated group. (**B**) Expression levels of caspase-4 protein analyzed by Western blot (*n* = 1). 1—*E. coli* LPS (1 µg/mL), 2—*K. japonica* LOS (1 µg/mL), 3—*K. japonica* LOS (10 µg/mL), 4—*K. japonica* LOS (1 µg/mL) + *E. coli* LPS (1 µg/mL), and 5—*K. japonica* LOS (10 µg/mL) + *E. coli* LPS (1 µg/mL).

In the final set of experiments, we evaluated the ability of *K. japonica* KMM 3897 LOS to induce IL-18 secretion (measured by enzyme-linked immunosorbent assay (ELISA)) and caspase-4 activation (determined by Western blot), both alone and in combination with *E. coli* LPS co-stimulation (Figure 9). Human PBMCs were stimulated for 18 h with *K. japonica* KMM 3897 LOS at concentrations of 1 and 10 μg/mL, and with *E. coli* LPS at a concentration of 1 μg/mL as a comparative control. In parallel, PBMCs were pretreated for 1 h with different concentrations (1 and 10 μg/mL) of *K. japonica* KMM 3897 LOS and then stimulated with *E. coli* LPS (1 μg/mL) for 18 h.

As shown in Figure 9, *K. japonica* KMM 3897 LOS demonstrated only marginal immunostimulatory activity, inducing minimal IL-18 secretion and caspase-4 activation compared with controls. More significantly, pretreatment experiments revealed potent dose-dependent inhibition of *E. coli* LPS-induced responses, with *K. japonica* LOS effectively suppressing both cytokine production and caspase-4 activation (Figure 9B, Figure S6 and Figure S7). Further investigation into its mechanism of action could provide valuable insights into its role in immune regulation.

## 3. Discussion

The discovery of microbial life thriving in diverse marine ecosystems worldwide has spurred the development of compelling hypotheses about the mechanisms that enable these microorganisms to endure extreme environmental conditions. Central to these hypotheses is the growing recognition that the chemical architecture of LPS in Gram-negative bacteria is critical to their ability to adapt and flourish in aquatic habitats [4]. Indeed, LPS undergoes significant structural modifications in response to environmental pressures, resulting in chemical features that are markedly distinct from those observed in terrestrial Gram-negative bacteria. For instance, the lipid A moiety of LPS in marine bacteria often exhibits a lower degree of acylation, typically being penta- or tetra-acylated. Additionally, the lipid A moiety of LPS from marine and extremophilic microorganisms tends to include desaturated acyl chains, shorter chain lengths, and increased branching. Furthermore, the phosphorylation level is often reduced, with some species expressing mono- or even non-phosphorylated lipid A molecules, further distinguishing them from their terrestrial counterparts [4,28,29,30,31,32,33,34,35,36,37,38,39]. The core oligosaccharide structures in marine Gram-negative bacteria are generally characterized by short chains and the presence of anionic functional groups [4,28,40,41,42,43]. Meanwhile, certain LPSs derived from marine bacteria exhibit remarkably low or even negligible immunostimulatory activity, a striking contrast with the potent inflammatory responses triggered by their pathogenic counterparts. This unique immunological neutrality, combined with the immense and largely untapped biodiversity of marine ecosystems, positions these molecules as exceptionally promising candidates for bioinspired therapeutics [4,5,6,7,8,9,15].

Given this, we report the structural characterization of the LOS isolated from the marine bacterium *K. japonica* KMM 3897, as determined through an integrated approach combining chemical analysis, NMR spectroscopy, and MALDI-TOF mass spectrometry. Briefly, the short core oligosaccharide moiety turned out to be a *mono*-phosphorylated trisaccharide containing 2-amino-2-deoxy-D-glucose, D-*glycero*-D-*manno*-heptose, and 3-deoxy-D-*manno*-oct-2-ulosonic acid. The penta-acylated lipid A was revealed to be composed of a glucosamine disaccharide backbone decorated by one or two phosphate groups and acylated by i11:0 (3-OH) as primary amide- and ester-linked fatty acids, and i11:0 (or 11:0) as a secondary acyl chain at position 2′ of the distal β-D-Glc*p*N. The tetra-acylated lipid A was revealed to be an isoform lacking one primary i11:0(3-OH) residue at position 3′ of the distal β-D-Glc*p*N. Minor hexa-acylated lipid A species, as well as one more minor penta-acylated species, were also detected.

From a biological perspective, we evaluated the immunostimulatory activity of *K. japonica* KMM 3897 LOS, which demonstrated markedly weaker cytokine induction in human PBMCs compared with *E. coli* LPS. Notably, *K. japonica* KMM 3897 LOS exhibited potent antagonistic activity against *E. coli* LPS-mediated toxicity in PBMCs, a characteristic previously documented for certain extremophilic and marine-derived LPSs [4,28,29,32,44]. This biological behavior may be attributed to the presence of hypo-acylated lipid A species in *K. japonica* KMM 3897 LOS, which not only minimizes pro-inflammatory immune activation but also effectively competes with pathogenic LPS for TLR4/MD-2 binding. Importantly, Western blot analysis revealed that *K. japonica* KMM 3897 LOS suppressed caspase-4 activation in *E. coli* LPS-treated PBMCs, demonstrating its protective anti-inflammatory properties.

Recent studies reveal intriguing differences in how caspase-4/11 and TLR4/MD-2 detect lipid A, challenging the assumption that both sensors share identical structural preferences [9,10,11]. While TLR4 strictly requires hexa- or hepta-acylated lipid A for activation, caspase-4/11 exhibits unexpected flexibility, responding to certain penta-acylated variants depending on the secondary acyl chain positioning (3′ versus 2′ on the distal β-D-Glc*p*N). Notably, some penta-acylated lipid A structures potently activate caspase-11 but weakly stimulate TLR4/MD-2, suggesting pathogen-specific immune evasion strategies [10,11]. Conversely, *Rhizobium* and *Rhodobacter* lipid As, despite being penta-acylated, inhibit caspase-11, highlighting how subtle structural variations (2′ secondary chains) can convert agonists into antagonists [10,11]. Further complexity arises in human macrophages, where caspase-4/5 activation requires lipid A and core oligosaccharide, unlike murine caspase-11 [11,18]. This species-specific requirement underscores the adaptability of inflammasome sensing. Moreover, caspase-4/11’s unique sensitivity to lipid A phosphate groups, rather than acyl chain number, contrasts sharply with TLR4’s narrow acylation dependence, broadening the immune system’s capacity to detect diverse LPS variants [10].

## 4. Materials and Methods

### 4.1. Materials

The organic solvents, inorganic acids, and salts were commercial products (Vekton, Russia). The Protein Plus molecular weight marker was purchased from Bio-Rad (Hercules, CA, USA). Acetyl chloride and trihydroxyacetophenone (THAP) were purchased from Merck (Darmstadt, Germany). For the protein detection by Western blotting, the following antibodies were used: caspase-4 (Cloud-Clone Corp., Wuhan, China, #PAB735Hu01, 1:1000), GAPDH (Cell Signaling Technology, Danvers, MA, USA, #2118T, 1:1000), and anti-rabbit IgG-HRP (Cell Signaling Technology, Danvers, MA, USA, #7074, 1:5000). For the cytokine detection, the following ELISA kits were used: TNF-α (Vector-Best, Novosibirsk, Russia, #8756), IL-1β (Vector-Best, Novosibirsk, Russia, #8766), IL-6 (Vector-Best, Novosibirsk, Russia, #8768), and IL-18 (Vector-Best, Novosibirsk, Russia, #8770).

### 4.2. Bacterial Strain, Isolation, and Purification of LOS, OS, and Lipid A

The bacterial strain *K. japonica* KMM 3897 was obtained from the Collection of Marine Microorganisms (KMM), the G.B. Elyakov Pacific Institute of Bioorganic Chemistry, the Far Eastern Branch of the Russian Academy of Sciences (Vladivostok, Russia). The bacteria were cultivated following a previously described method [45]. The crude LOS was extracted from dried bacterial cells (8 g) using the phenol/chloroform/petroleum ether (PCP) method [24], yielding 145 mg of material. For further analysis, a 50 mg aliquot of the LOS sample was treated with 12.5% aqueous ammonium hydroxide at 37 °C for 16 h to achieve de-O-acylation, followed by lyophilization. The de-O-acylated LOS was then subjected to alkaline hydrolysis by heating with 4 M potassium hydroxide (1.0 mL) at 120 °C for 16 h. The reaction mixture was neutralized with 2 M hydrochloric acid (to pH ~6) and extracted three times with chloroform. The aqueous layer was desalted in a Toyopearl HW-40 column (120 × 1.5 cm, Tosoh Bioscience, Tokyo, Japan) using 0.1% aqueous acetic acid as the eluent, yielding 6.2 mg of OS. Elution was monitored using a differential refractometer (Knauer, Griesheim, Germany). To investigate the lipid A structure, a separate 50 mg aliquot of pure LOS was hydrolyzed in acetate buffer (pH 4.4) at 100 °C for 3 h. The hydrolysate was mixed with chloroform and methanol to achieve a final ratio of chloroform/methanol/hydrolysate of 2:2:1.8 (*v*/*v*/*v*). After shaking and centrifugation, the organic phase containing the lipid A fraction was collected and washed with the aqueous phase of a freshly prepared Bligh–Dyer mixture (chloroform/methanol/water (2:2:1.8)). The chloroform phase was dried (5.4 mg) and analyzed by MALDI-TOF MS.

### 4.3. Chemical Analysis of LOS

Monosaccharides were analyzed as acetylated methyl glycosides, using appropriate authentic standards as references. For methanolysis, a LOS sample (1 mg) was treated with 1.25 M acetyl chloride in methanol (1 mL) at 80 °C for 16 h. The resulting mixture was extracted three times with hexane to separate fatty acid methyl esters (FAMEs) from O-methyl glycosides. The hexane phase was dried and analyzed by GC-MS. The remaining methanolic phase was dried, and the O-methyl glycosides were acetylated with acetic anhydride in pyridine at room temperature for 16 h. The absolute configurations of the sugar residues were determined by GC-MS analysis of the acetylated (*S*)-2-butyl glycosides, as described in [46]. All derivatives were analyzed using a Hewlett Packard 5890 chromatograph (Conquer Scientific, Poway, CA, USA) coupled with a Hewlett Packard 5973 mass spectrometer (USA) and an HP-5MS capillary column. Acetylated methyl glycosides and acetylated (*S*)-2-butyl glycosides were analyzed using the following temperature program: 150 °C for 3 min, 150 °C → 280 °C at 3 °C/min, and 280 °C for 10 min. The following temperature program was employed for the FAME analysis: 120 °C for 3 min, 120 °C → 280 °C at 4 °C/min, and 280 °C for 10 min. To obtain the dephosphorylated LOS, the sample (1 mg) was treated with aq 48% hydrofluoric acid (0.1 mL, 4 °C, 16 h). The hydrofluoric acid was evaporated, and the modified LOS was analyzed as described above.

### 4.4. NMR Spectroscopy and MALDI-TOF MS Analysis

The OS was analyzed by ^1^H and ^13^C NMR spectroscopy using a Bruker Avance-III spectrometer (700.13 MHz for ^1^H, 176.04 MHz for ^13^C; Bruker, Karlsruhe, Germany). Spectra were acquired at 37 °C in 99.95% D_2_O, with acetone (δ_C_ 31.45 and δ_H_ 2.225) as an internal reference standard. The ^1^H,^1^H-TOCSY and ^1^H,^1^H-ROESY spectra were recorded with a 180 ms duration of MLEV-17 spin-lock and a 300 ms mixing time, respectively. ^1^H,^13^C-HMBC and ^1^H,^31^P-HMBC were optimized for an 8 Hz long-range constant. MALDI-TOF mass spectra were acquired with an ULTRAFLEX III (Bruker, Karlsruhe, Germany) mass spectrometer. A solution of THAP in methanol/aq 0.1% trifluoroacetic acid/acetonitrile (7:2:1, *v*/*v*/*v*) at a concentration of 75 mg/mL was used as the matrix. The lipid A sample was dissolved in chloroform/methanol (3:1).

### 4.5. Human PMBCs’ Isolation, Stimulation, and Cytokine Measurement

PBMCs were isolated from healthy adult volunteers after obtaining written informed consent using density gradient centrifugation and cultured as previously described [39]. For the stimulation experiments, cells were seeded in 24-well plates (2.5 × 10^5^ cells/well) and treated with LOS from either *K. japonica* KMM 3897 (10, 100 ng/mL or 1, 10 µg/mL) or commercial *E. coli* O111:B4 (10 ng/mL or 1 µg/mL) for 18 h before supernatant collection for ELISA. In competition assays, PBMCs were pre-incubated with *K. japonica* LOS (10, 100 ng/mL or 1, 10 µg/mL) for 1 h, followed by stimulation with *E. coli* O111:B4 LPS (10 ng/mL or 1 µg/mL) for 18 h. The cytokine levels (TNF-α, IL-1β, IL-6, and IL-18) in the supernatants were quantified using manufacturer-optimized ELISA kits. The results are expressed as the % change in cytokines’ production relative to the negative control (100%).

### 4.6. Protein Preparation and Western Blotting

PBMCs were seeded at 1 × 10^6^ cells/well in 6 cm Petri dishes (5 mL/dish) and incubated overnight before treatment with either *K. japonica* KMM 3897 LOS (1 and 10 µg/mL) or *E. coli* O111:B4 LPS (1 µg/mL) for 18 h at 37 °C with 5% CO_2_. For competition assays, PBMCs were pretreated with *K. japonica* LOS (1 and 10 µg/mL) for 1 h prior to stimulation with *E. coli* O111:B4 LPS (10 ng/mL or 1 µg/mL) for 18 h. Following incubation, the cells were harvested by scraping, washed, and lysed using Chaps Cell Extract Buffer (Cell Signaling Technology, Danvers, MA, USA). The protein extracts were separated by electrophoresis, transferred to PVDF membranes, and probed with primary and secondary antibodies (Cell Signaling Technology, Danvers, MA, USA; Cloud-Clone Corp., Wuhan, China), with signal detection performed using a ChemiDoc MP Imaging System (Bio-Rad, Hercules, CA, USA).

### 4.7. Data Analysis

The results are expressed as a % relative to the negative control (100%). Each bar represents the mean ± SEM of three independent replicates (*n* = 3). Statistical significance was determined by one-way ANOVA followed by Tukey’s Honest Significant Difference test. A *p*-value < 0.05 was considered statistically significant.

## 5. Conclusions

In conclusion, our preliminary results align with the established research while highlighting the potential of marine microorganisms as sources of immune response modifiers. The dual capacity to simultaneously inhibit TLR4/MD-2 and caspase-4/5 activation could provide instruments to control acute inflammatory responses and reduce LPS-induced toxic effects. While these preliminary findings demonstrate applicable immunomodulatory properties, their therapeutic potential requires further validation. Future studies should address comparative analysis across phylogenetically diverse marine Gram-negative bacterial strains, in vivo efficacy testing, and detailed mechanistic studies elucidating structure–activity relationships.

## Figures and Tables

**Table 1:** ^1^H and ^13^C chemical shifts of the OS from *K. japonica* 3897 (δ, ppm).

Sugar Residue	H-1 C-1	H-2 C-2	H-3eq,ax C-3	H-4 C-4	H-5 C-5	H-6a,b C-6	H-7a,b C-7	H-8a,b C-8
→6)-α-D-Glc*p*N1P	5.74	3.45	3.93	3.52	4.12	4.27, 3.89		
**A**	92.9	55.2	70.6	70.8	73.7	70.1		
→6)-β-D-Glc*p*N4P-(1→	4.88	3.16	3.91	3.95	3.77	3.79, 3.63		
**B**	100.1	56.7	72.8	75.3	74.9	63.8		
→5)-α-Kdo*p*4P-(1→			2.29, 2.12	4.60	4.34	3.86	3.87	3.91, 3.69
**C**	173.4	100.3	35.0	70.8	73.7	70.2	73.2	64.4
→4)-α-D,D-Hep*p*-(1→	5.13	4.11	4.11	4.07	4.30	4.04	3.76, 3.88	
**D**	101.0	70.8	70.1	78.1	73.4	72.9	63.5	
β-D-Glc*p*N-(1→	4.92	3.14	3.71	3.52	3.54	3.93, 3.78		
**E**	98.8	57.0	73.2	70.8	77.6	61.5		

**Table 2:** The main ion peaks observed in the MALDI-TOF MS spectrum reported in Figure 4, the predicted masses, and the proposed interpretation of the substituting fatty acids, and phosphate (P), on the lipid A backbone. The observed masses listed in the table are compared with the calculated monoisotopic mass (predicted mass (Da)) of each ion based on the proposed lipid A structures.

Lipid A (Figure 4)		
Predicted Mass (Da)	Observed Ion Peaks (*m*/*z*)	Proposed Composition
1544.9	1543.4	HexN^2^, P^2^, [i11:0(3-OH)]^4^, i11:0 (or 11:0), 9:0
1404.8	1403.4	HexN^2^, P^2^, [i11:0(3-OH)]^4^, i11:0 (or 11:0)
1348.8	1347.4	HexN^2^, P^2^, [i11:0(3-OH)]^4^, 7:0
1324.9	1323.4	HexN^2^, P, [i11:0(3-OH)]^4^, i11:0 (or 11:0)
1220.7	1219.3	HexN^2^, P^2^, [i11:0(3-OH)]^3^, i11:0 (or 11:0)
1140.7	1139.4	HexN^2^, P, [i11:0(3-OH)]^3^, i11:0 (or 11:0)

## Data Availability

Dataset available on request from the authors.

## Supplementary Materials

The supporting information can be downloaded at https://www.mdpi.com/article/10.3390/md23090345/s1, Figure S1. Silver-stained electropherogram: lane 1—*E. coli* 0111:B4 LPS 8 μg, lane 2—*K. japonica* KMM 3897 8 μg; Figure S2. ^1^H NMR spectrum of the OPS from *K. japonica* KMM 3897; Figure S3. DEPT-135 spectrum of the OS from *K. japonica* KMM 3897; Figure S4. ^1^H, ^1^H-COSY spectrum of the OS from *K. japonica* KMM 3897; Figure S5. ^1^H, ^1^H-TOCSY spectrum of the OS from *K. japonica* KMM 3897; Figure S6. Expression levels of Caspase 4 protein analyzed by western blot. 1—*E. coli* LPS (1 μg/mL), 2—*K. japonica* LOS (1 μg/mL), 3—*K. japonica* LOS (10 μg/mL), 4—*K. japonica* LOS (1 μg/mL) + *E. coli* LPS (1 μg/mL), 5—*K. japonica* LOS (10 μg/mL) + *E. coli* LPS (1 μg/mL); Figure S7. Expression levels of GAPDH protein analyzed by western blot. 1—*E. coli* LPS (1 μg/mL), 2—*K. japonica* LOS (1 μg/mL), 3—*K. japonica* LOS (10 μg/mL), 4—*K. japonica* LOS (1 μg/mL) + *E. coli* LPS (1 μg/mL), 5—*K. japonica* LOS (10 μg/mL) + *E. coli* LPS (1 μg/mL).
