# Supplementary material for: Structural and Immunological Insights into the Lipooligosaccharide of the Marine Bacterium Kangiella japonica KMM 3897
Source: Mar Drugs. 2025 Aug 28;23(9):345. doi: 10.3390/md23090345 (PMC12472108; doi:10.3390/md23090345)
Supplement: Supplementary file 1 [file marinedrugs-23-00345-s001.zip › marinedrugs-3802499-supplementary.pdf]

Structural and Immunological Insights into the Lipooligosaccharide of Marine Bacterium *Kangiella japonica* KMM 3897

Alina P. Filshtein[1,†], Vlada S. Belova[1,2], Alexandra S. Kuzmich[1], Lyudmila A. Romanenko[1] and Maxim S. Kokoulin[1,†*]

[1] G.B. Elyakov Pacific Institute of Bioorganic Chemistry, Far Eastern Branch, Russian Academy of Sciences, 159/2, Prospect 100 let Vladivostoku, Vladivostok, 690022, Russia; alishichka@mail.ru; vladabelova306@gmail.com; assavina@mail.ru; lro@piboc.dvo.ru; maxchem@mail.ru

[2] Far Eastern Federal University, 10 Ajax Bay, Russky Island, Vladivostok, 690922, Russia; vladabelova306@gmail.com

* Correspondence: maxchem@mail.ru

†These authors contributed equally to this work

Figure S1. Silver-stained electropherogram: lane 1 - *E. coli* 0111:B4 LPS 8 µg, lane 2 – *K. japonica* KMM 3897 8 µg

Figure S2. ^{1}H NMR spectrum of the OPS from *K. japonica* KMM 3897

Figure S3. DEPT-135 spectrum of the OS from *K. japonica* KMM 3897

Figure S4. ^{1}H, ^{1}H-COSY spectrum of the OS from *K. japonica* KMM 3897

Figure S5. ^{1}H, ^{1}H-TOCSY spectrum of the OS from *K. japonica* KMM 3897

Figure S6. Expression levels of Caspase 4 protein analyzed by western blot. 1 - E. coli LPS (1 µg/mL), 2 - K. japonica LOS (1 µg/mL), 3 - K. japonica LOS (10 µg/mL), 4 - K. japonica LOS (1 µg/mL) + E. coli LPS (1 µg/mL), 5 - K. japonica LOS (10 µg/mL) + E. coli LPS (1 µg/mL)

Figure S7. Expression levels of GAPDH protein analyzed by western blot. 1 - E. coli LPS (1 µg/mL), 2 - K. japonica LOS (1 µg/mL), 3 - K. japonica LOS (10 µg/mL), 4 - K. japonica LOS (1 µg/mL) + E. coli LPS (1 µg/mL), 5 - K. japonica LOS (10 µg/mL) + E. coli LPS (1 µg/mL)

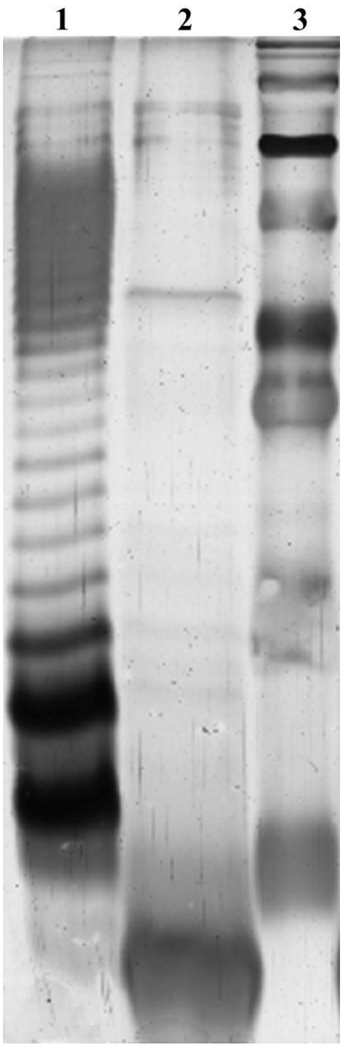

Figure S1. Silver-stained electrophoregram: lane 1 - *E. coli* 0111:B4 LPS 8 µg, lane 2 – *K. japonica* KMM 3897 8 µg

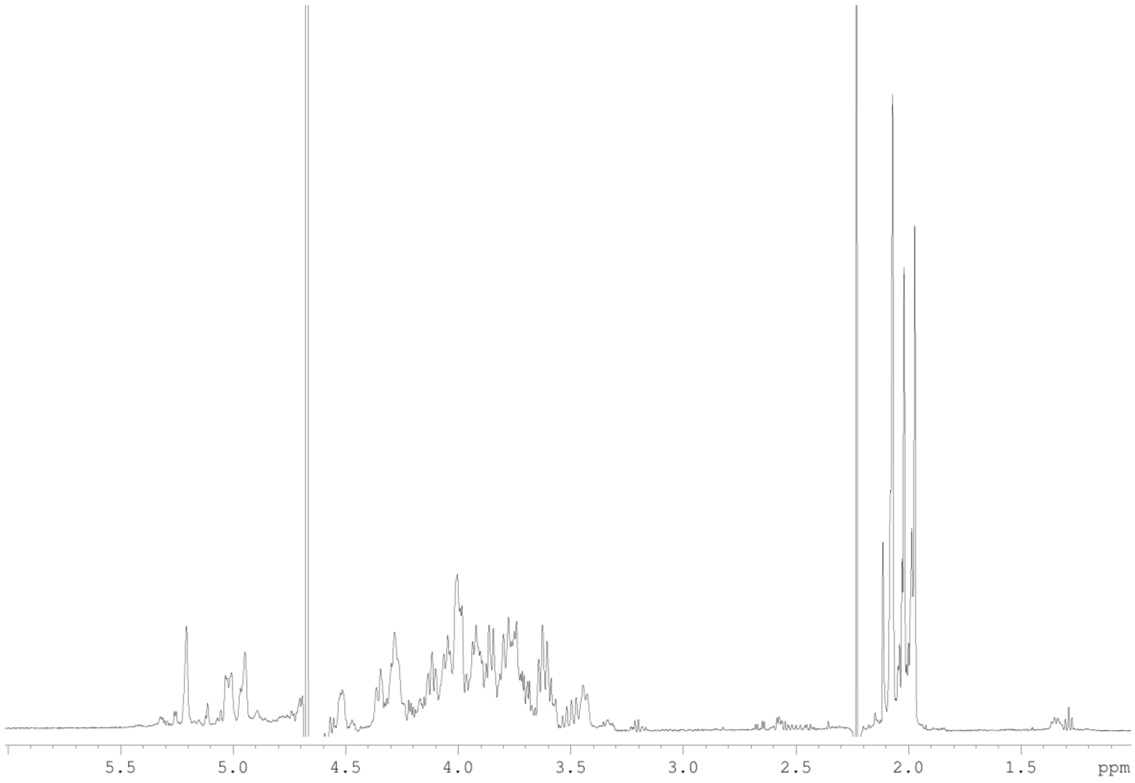

Figure S2. ^{1}H NMR spectrum of the OPS from *K. japonica* KMM 3897

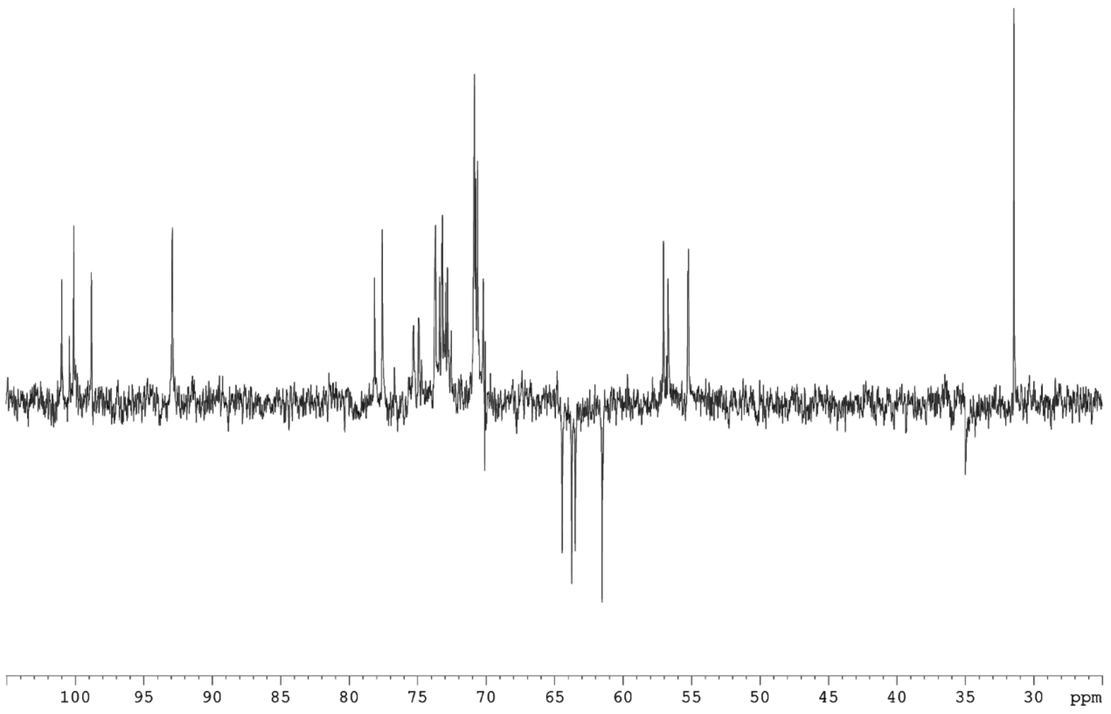

Figure S3. DEPT-135 spectrum of the OS from *K. japonica* KMM 3897

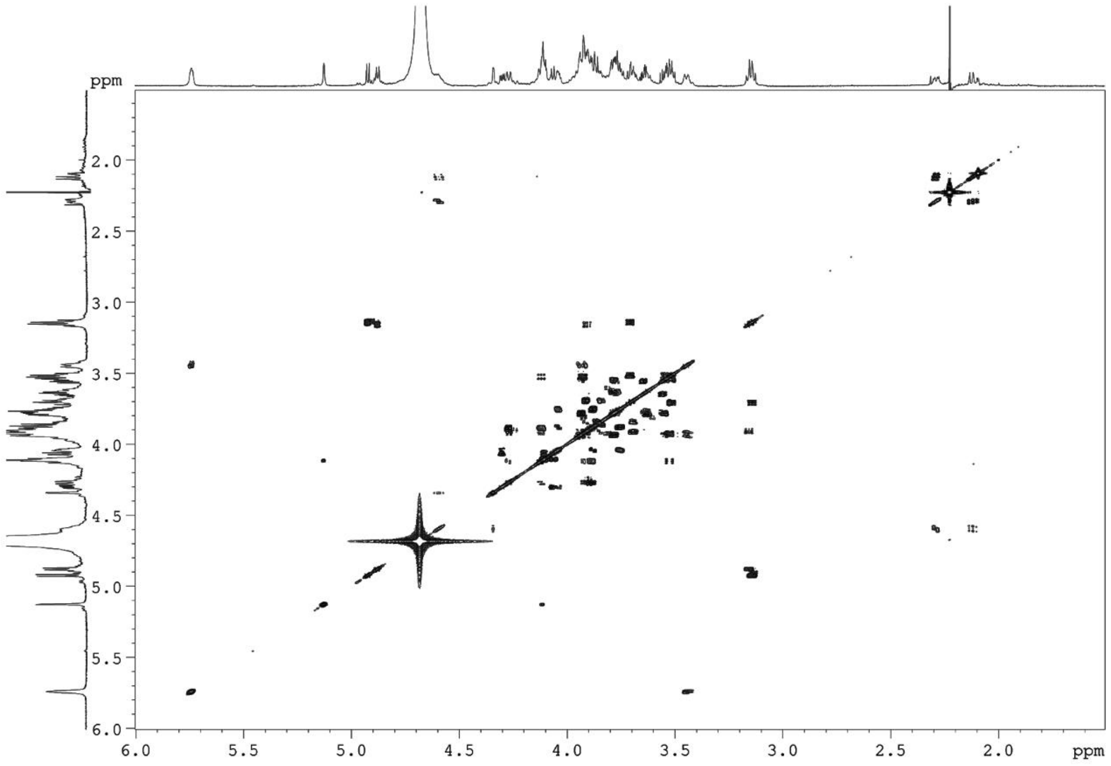

Figure S4. ^{1}H, ^{1}H-COSY spectrum of the OS from *K. japonica* KMM 3897

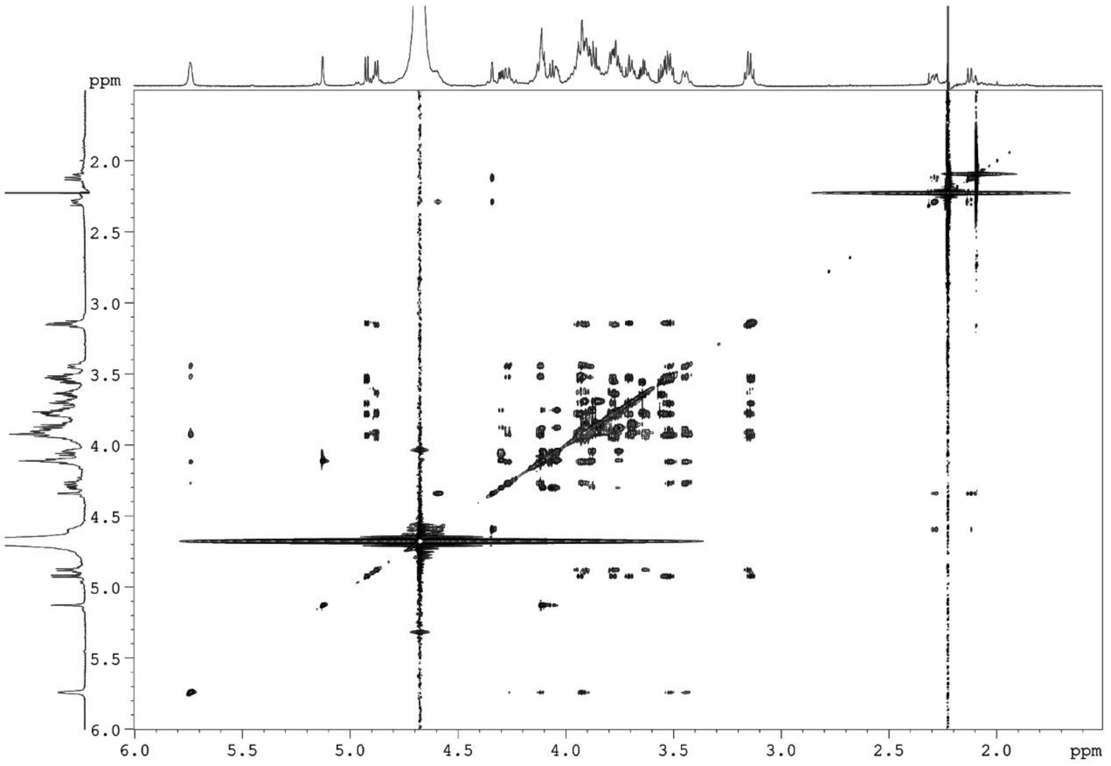

Figure S5. ^{1}H, ^{1}H-TOCSY spectrum of the OS from *K. japonica* KMM 3897

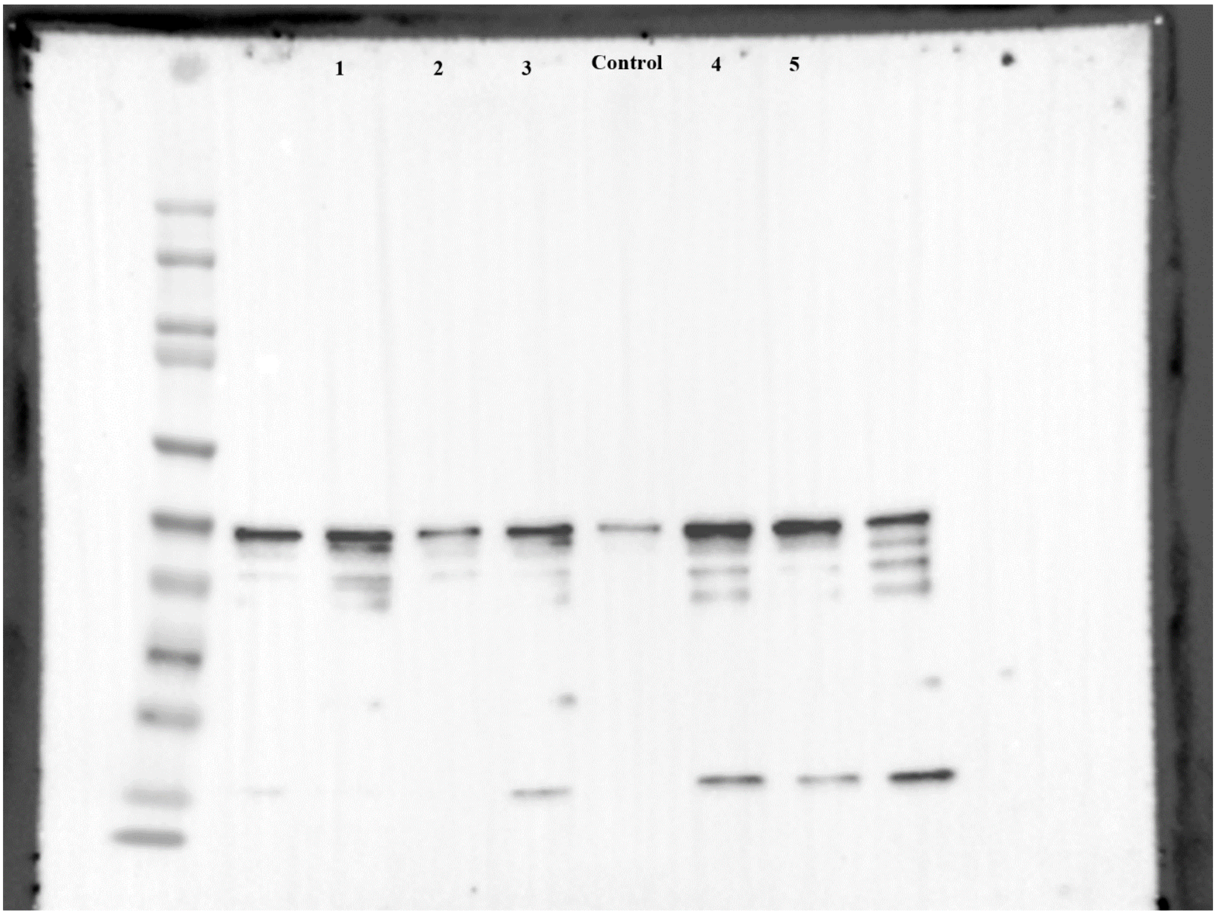

Figure S6. Expression levels of Caspase 4 protein analyzed by western blot. 1 - E. coli LPS (1 µg/mL), 2 - K. japonica LOS (1 µg/mL), 3 - K. japonica LOS (10 µg/mL), 4 - K. japonica LOS (1 µg/mL) + E. coli LPS (1 µg/mL), 5 - K. japonica LOS (10 µg/mL) + E. coli LPS (1 µg/mL)

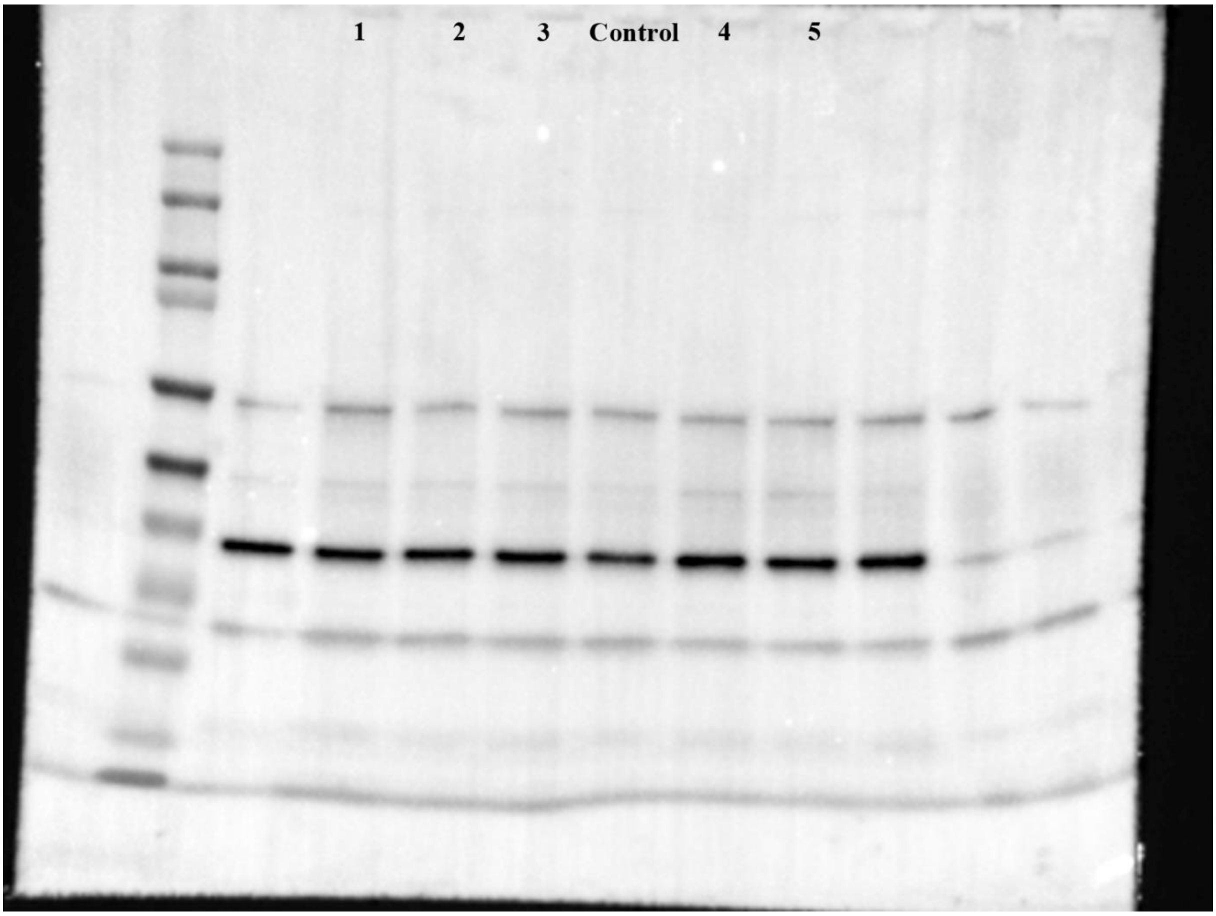

Figure S7. Expression levels of GAPDH protein analyzed by western blot. 1 - E. coli LPS (1 µg/mL), 2 - K. japonica LOS (1 µg/mL), 3 - K. japonica LOS (10 µg/mL), 4 - K. japonica LOS (1 µg/mL) + E. coli LPS (1 µg/mL), 5 - K. japonica LOS (10 µg/mL) + E. coli LPS (1 µg/mL)
